# Impact of Social Jetlag on Weight Change in Adults: Korean National Health and Nutrition Examination Survey 2016–2017

**DOI:** 10.3390/ijerph17124383

**Published:** 2020-06-18

**Authors:** Jin Hwa Kim, Young Sang Lyu, Sang Yong Kim

**Affiliations:** Department of Endocrinology and Metabolism, Chosun University Hospital, Gwangju 61453, Korea; endocrine@chosun.ac.kr (J.H.K.); csh2014059@csuh.co.kr (Y.S.L.)

**Keywords:** social jetlag, weight gain, circadian misalignment

## Abstract

Social jetlag, the circadian misalignment reflecting the discrepancy between the circadian clock and social clock, has been implicated in weight-related issues. The objective of the present study was to determine whether there was an association between social jetlag and body weight change among adults in a large, nationally representative general population. This study was based on data from the Korean National Health and Nutrition Examination Survey, conducted during 2016–2017 by the Korean Ministry of Health and Welfare. Of the 16,277 participants, data from 8295 adults were included in the analysis. Men with social jetlag > 2 h had a significantly higher risk of weight gain (odd ratios (OR): 1.787; 95% confident interval (CI): 1.192–2.679) than those with social jetlag < 1 h, after adjustment for age, sociodemographic factors, lifestyle behaviors, chronic disease, obesity and average sleep duration. Women with weight gain had a higher social jetlag (>2 h), and women with social jetlag > 2 h had a higher proportion of weight gain. However, we did not find a significant association of social jetlag with weight gain after adjusting for confounding factors in women. There was no significant association between social jetlag and weight loss in men and women. Higher social jetlag was independently associated with an increased risk of weight gain in men. We propose that social jetlag may contribute to the obesogenic tendency in men, and that there is a potential for body weight to be managed with a circadian approach.

## 1. Introduction

All organisms on Earth are governed by rhythms at all times. These biological rhythms are maintained by an endogenous circadian clock, which consists of the central clock in the suprachiasmatic nucleus (SCN) of the hypothalamus and peripheral clocks [1,2]. This clock sets up its own program and builds up networks for circadian timekeeping. The circadian clock regulates the cellular, physiological and metabolic systems to synchronize biological cycles with environmental cycles [3]. The flow of life in modern society is too complex and irregular to fit every endogenous circadian clock. Therefore, we can often become a ‘time traveler’, traveling across the body’s biological time zones set by social schedules.

Social jetlag is a chronic jetlag-like phenomenon, or more specifically, the travel between the ‘time zone’ on workdays (time by socially imposed schedules) and free days (time regulated by the circadian clock) [4]. Social jetlag can result in chronic and ongoing circadian misalignment because of a mismatch between circadian and external timing, which may contribute to metabolic risk by desynchronizing the temporal organization of various metabolic processes. Recently, interest in the impact of circadian misalignment on health issues has grown [5,6,7].

Obesity is associated with metabolic diseases, and body weight control is a major concern in general public health [8,9]. As the prevalence of obesity increases, the identification of individuals at risk for weight gain would be valuable for initiating earlier precision strategies to prevent the development of obesity and improve overall public health.

Several studies suggest that circadian misalignment—as with social jetlag—may contribute to weight-related issues. Roenneberg et al. demonstrated that social jetlag was positively associated with increased body mass index, and the risk of moving from being overweight to obese in particular [10]. Another study suggested that social jetlag was associated with a higher risk of being overweight and related metabolic complications [11]. Parsons et al. also showed that individuals with high social jetlag had an increased risk of metabolically unhealthy obesity [12].

However, to our knowledge, there are no large general population-based reports investigating the influence of social jetlag on body weight change in adults. The objective of the present study was to determine whether there is an association between social jetlag and body weight change among adults in a nationally representative large general population.

## 2. Materials and Methods

### 2.1. Study Population

Data from the Korean National Health and Nutrition Examination Survey (KNHANES) VII, conducted by the Korean Ministry of Health and Welfare in 2016–2017, were used for this study. The KNHANES is a cross-sectional survey of a nationally representative sample of noninstitutionalized civilians residing in Korea. This population-based survey used a stratified, multistage, clustered probability sampling design. The sampling plan was based on data from household registries, including geographic area, sex and age groups. The data in KNHANES consist of a health interview, a nutrition interview and a health examination survey conducted by trained investigators. All of the participants signed an informed consent form. This study was approved by the Korea Centers for Disease Control and Prevention Institutional Review Board (approval no. 2013-12Exp-03-5C).

Of the 16,277 participants in the 2016–2017 survey, we used data collected from 12,773 participants who were ≥20 years. We excluded participants with missing data on sleep status (*n* = 1287) and on weight change for the previous 1 year (*n* = 34). Subjects were also excluded if they had a history of cancer (*n* = 602), chronic kidney disease (*n* = 38) or liver cirrhosis (*n* = 25); were pregnant (*n* = 64); had fasted <8 h (*n* = 316); performed shift work (night shift work, day–night rotating work, 24 h rotating work, split work, irregular work schedules and others) (*n* = 479); or missing or inadequate data (*n* = 1633). Ultimately, data for 8295 (men 3417, women 4878) were retained for analysis.

### 2.2. Measurement and Classification of Variables

Height and body weight were measured using a portable stadiometer (SECA 225, SECA Deutschland, Hamburg, Germany) and a balanced scale (GL-6000-20, CAS KOREA, Seoul, Korea). The mean value of two separate blood pressure (BP) measurements after 5 min of rest in the sitting position was used for analysis. Venous blood samples were performed after a minimum fasting time of 8 h.

Self-reported questionnaires consisted of smoking status, alcohol consumption, family income, occupation, education level, residential area, regular exercise, and total energy, carbohydrate and fat intake. Occupation was categorized as none, nonmanufacturing (government officials, managers, professionals and office workers) and manufacturing (services, sales, agriculture and fishery industries, and factory workers). Residential area was categorized according to the Korean administrative district as an urban or rural area. Regular exercise was indicated as ‘yes’ when the subject performed aerobic exercise on a regular basis. The total fat, carbohydrate and energy intake was calculated as a sum of foods containing fat, carbohydrate and energy from a 24 h recall nutrition survey questionnaire.

Subjects were asked to indicate any changes in body weight with the question “Were there any changes in weight compared to the previous year?”. For the question about weight change, the answer was categorized as ‘no changes (weight changes of less than 3 kg)’, ‘weight loss’ and ‘weight gain’.

Subjects were also asked to recall sleep time and wake time on weekdays (working days) and weekends (free days) with the questions “What time do you sleep and wake on weekdays (or working days)?” and “What time do you sleep and wake on weekends (or free day and the day before free day)?”. Social jetlag was calculated following the protocol established by Wittman et al. [4], with the absolute difference in hours in the midpoint of sleep between weekends and weekdays. For data analysis, subjects were categorized into three groups according to social jetlag: <1 h, 1–2 h and >2 h. Sleep duration was calculated as the average sleep duration on weekdays and weekends.

Obesity was defined according to the criteria recommended by the Korean Society for the Study of Obesity, with BMI ≥ 25.0 kg/m^2^ defined as obese [13]. Diabetes was defined as undergoing treatment or being diagnosed by a physician, or FPG ≥ 126 mg/dL or HbA1c ≥ 6.5%. Hypertension was defined as undergoing treatment or being diagnosed by a physician, or systolic BP ≥ 140 mmHg or diastolic BP ≥ 90 mmHg.

### 2.3. Statistical Analysis

We used complex sample analysis and applied to the KNHANES data to weight all values following a tutorial for statistical analysis from the Korea Centers for Disease Control and Prevention. Continuous variables were reported as mean ± SD, and categorical variables were reported as weighted percentages. The comparisons among groups were performed using ANOVA for continuous variables, and the Chi-square tests was used for categorical variables. We used the same covariates for this analysis (Table 1, Table 2, Table 3 and Table 4). Multivariate logistic regression analyses were used separately to analyze the association between social jetlag and weight change. We evaluated the odds ratio after adjusting for confounding factors in a group stratified by two groups with weight change, according to sex. The covariates included in the model were factors that were found to be significantly different in the comparison of groups, as follows: in men, covariates included age, sociodemographic factors (family income, occupation and education), lifestyle behaviors (smoking status, alcohol consumption, regular exercise, total energy intake and fat intake), chronic disease (diabetes and hypertension), obesity and sleep duration. In women, covariates included age, sociodemographic factors (residential area, family income, occupation and education), lifestyle behaviors (smoking status, alcohol consumption, regular exercise, total energy intake, carbohydrate intake and fat intake), chronic disease (diabetes and hypertension), obesity and sleep duration.

We analyzed using SPSS software version 18.0 (SPSS Inc., Chicago, IL, USA) for statistical analyses, and a *p* value of <0.05 was considered to indicate statistical significance.

## 3. Results

### 3.1. Characteristics of the Study Subjects

The basic characteristics of weight change in men are shown in Table 1. The proportions of men with weight loss and weight gain were 13.1% (448/3417) and 20.1% (686/3417), respectively. Men with weight gain were younger and more likely to have higher BMI and fat intake. Subjects with weight gain were more likely to be non-smokers, drink alcohol and have nonmanufacturing occupations, and were less likely to have lower education levels, hypertension and diabetes. Men with weight loss or gain participated in more regular exercise. No significant differences were found in family income, residential area, total energy intake, carbohydrate intake or sleep duration among the subgroups. Men with weight gain were more likely to have higher social jetlag and >2 h of social jetlag than those with no weight change or weight loss.

Table 2 shows the basic characteristics of the different weight changes in women. The proportions of women with weight loss and weight gain were 11.6% (568/4878) and 27.5% (1341/4878), respectively. Women with weight gain were younger and more likely to have higher BMI and fat intake. Subjects with weight gain were more likely to drink alcohol, have a higher family income and have nonmanufacturing occupations, and were less likely to have a lower education level, hypertension and diabetes. Women with weight gain were more likely to be non-smokers and have higher carbohydrate intake than those with weight loss. No significant differences were found in residential area, regular exercise, total energy intake or sleep hours among the subgroups. Women with weight gain were more likely to have higher social jetlag and >2 h of social jetlag than those with no weight change or weight loss.

### 3.2. Comparison of Clinical Characteristics and Weight Change Status among Social Jetlag Subgroups

Table 3 shows the characteristics of the male subjects stratified into three groups by social jetlag. Men with higher social jetlag were younger and were more likely to smoke, drink alcohol, have a higher family income, have a nonmanufacturing occupation, reside in urban areas, exercise regularly, have higher total energy and fat intake, and sleep ≥7 h/night. In addition, men with higher jetlag were less likely to have lower education levels, hypertension and diabetes. The proportion of weight gain differed significantly between the subgroups, and was higher with social jetlag: this proportion was 19.1% for 1 h, 29.2% for 1–2 h, and 43.7% for >2 h. However, the proportion of weight loss was relatively similar between the subgroups (13.5% for 1 h, 12.0% for 1–2 h and 12.5% for >2 h).

Table 4 shows the characteristics of the female subjects stratified into three groups by social jetlag. Women with higher social jetlag were younger, had lower BMI and were more likely to smoke, drink alcohol, have a higher family income, have a nonmanufacturing occupation, reside in urban areas, exercise regularly, have higher total energy and fat intake and sleep ≥7 h/night. In addition, women with higher jetlag were less likely to have lower education levels, hypertension and diabetes. The proportion of weight gain differed significantly between the subgroups, and was higher with social jetlag: this proportion was 27.0% for 1 h, 31.4% for 1–2 h and 41.5% for >2 h. However, the proportion of weight loss was relatively similar between the subgroups (12.3% for 1 h, 11.1% for 1–2 h and 12.2% for >2 h).

### 3.3. Relationship between Social Jetlag and Weight Change

Table 5 shows the results of the logistic regression analyses designed to evaluate the relationship between social jetlag and weight change. Men with social jetlag of >2 h had a significant association with weight gain (odd ratios (OR): 1.787; 95% confident interval (CI): 1.192–2.679) compared with those with social jetlag of <1 h after adjustment for age, sociodemographic factors, lifestyle behaviors, chronic disease, obesity and sleep duration. However, there were no significant associations between social jetlag and weight loss in men.

In women, there was no significant association between social jetlag and weight gain after adjustment for confounding factors. Furthermore, no significant association of social jetlag with weight loss was found in women.

### 3.4. Stratified and Sensitivity Analyses

To evaluate whether higher social jetlag was consistently related to an increased risk of weight gain in men, we searched for interactions between social jetlag and covariates, and performed multivariate logistic regression analysis in each subgroup. There was no significant interaction between social jetlag and covariates in men (for all, *p* > 0.05).

## 4. Discussion

In this study, social jetlag (>2 h) was significantly associated with weight gain in men after adjustment for multiple potential confounding variables. Women with weight gain had higher social jetlag (>2 h), and women with social jetlag >2 h had a higher proportion of weight gain. However, we did not find a significant association of social jetlag with weight gain after adjusting for confounding factors in women. There was no significant association between social jetlag and weight loss in men and women. To our knowledge, this is the first large population-based study assessing the potential effects of social jetlag on body weight change. Our results suggest obesogenic effects of circadian misalignment on weight change in men.

Because this study had a cross-sectional design, determining the exact biological mechanism underlying our findings was not possible, but we propose several possibilities.

Social jetlag, or rapid traveling across a biologically programmed time zone, can be a challenge for the biological clock because it needs to immediately adapt to external time. The circadian clock comprises a self-sustaining interlocked network of transcriptional–translational feedback loops of the products of a panel of clock genes (including *CLOCK, BMAL1, PER1/2, CRY1/2* and *REV-ERB-a*) [1,2,3]. A greater degree of social jetlag results in a longer overall duration of exposure to circadian misalignment because of the immediate failure to adjust to external social time, which can lead to disruption of the coordination of various hormonal actions, physiology and metabolism, which can induce anabolic shifts in energy homeostasis.

Adipose tissue plays a crucial role in energy homeostasis, and timing is a crucial aspect in adipose tissue metabolism [14]. Leptin increases the activation of the sympathetic nervous system and thermogenesis, which consumes energy as heat [15]. Leptin sensitization also promotes energy expenditure through hypocretin signaling [16]. Leptin receptors are found in the SCN, and leptin release is controlled by the circadian cycle, whose levels peak at night [17]. The mistiming of the circadian clock due to social jetlag can affect leptin secretion and activation, which can lead to the disruption of energy expenditure and therefore result in a decrease in the basal metabolic rate, which can affect obesogenic tendencies. However, further studies are required because associations between leptin and metabolic disorders are rather complex and non-linear, and might be gender specific.

Glucocorticoids can also play a potential role in mediating the association between social jetlag and weight gain. The circadian rhythm of glucocorticoids is related to adipose tissue homeostasis [18]. Circadian misalignment can affect the secretion of glucocorticoids during external time. These effects can bring about the anabolic tendency of the metabolism, which can lead to subsequent weight gain. A recent study demonstrated that salivary glucocorticoid levels in the morning were higher in people with social jetlag ≥2 h than in people with ≤1 h [19].

With regard to energy metabolism, melatonin modulates the sympathetic nervous system, and acts directly on adipose tissue [20]. Melatonin supplementation prevented gains of body weight and fat depot mass, as well as adipocyte hypertrophy in an obesity model [21]. Oral supplementation in humans with melatonin deficiency increases the volume and activity of brown adipose tissue [22]. Melatonin, the synthesis and release of which is stimulated by darkness and suppressed by light, plays a crucial role in maintaining and adjusting body rhythm [23]. Exposure to light at programmed circadian nighttime causes the melatonin level to decrease. Appropriate timing is also important for the sensitivity of the organ to melatonin [24]. Phillips et al. reported the interindividual variability of the response of the human circadian system to evening light [25].

In addition, circadian rhythm disruption can alter the circadian clocks of intestinal microbiota, leading to a change in the intestinal microbiota community’s composition, which may have implications for weight gain [26]. Experimental circadian misalignment led to changes in the presence of bacterial communities that can promote increased energy absorption from food ingestion, and subsequent increases in energy balance [27].

These numerous exposures to an environment with a tendency to store energy and obesogenic effects might be cumulative and remembered. This repetitive process, even if repeated every week, could lead to a reprogramming of the biological rhythm to a ‘weight gain-prone phenotype’, with a greater tendency and more susceptibility to weight gain. Furthermore, this reprogramming might result in the evolution of the circadian clock gene to optimally exploit these changes to establish a more weight gain-prone program. Additional studies are needed to explore the exact causal relationships in more detail.

In the present study, it is unclear why social jetlag is differentially related to weight gain according to sex, occurring only in men. Sex differences in the circadian timing systems have been reported [28]. Several studies have demonstrated that estrogens regulate the oscillation of circadian genes in various organs [29,30]. The potential protective role of estrogen against disruptions in circadian rhythm has been reported [31]. Baron et al. reported that circadian alignment was associated with percent body fat in men only [32].

Health-related behaviors, socioeconomic status and sleep hours were considerable confounders of the association between social jetlag and weight gain in this study. Populations with higher social jetlag may have more unhealthy behaviors. Previous studies found that social jetlag is associated with the inappropriate timing of food intake, and high-fat feeding, smoking, alcohol use and lower levels of physical activity [33]. Unhealthy behaviors, lower socioeconomic status, obesity status and insufficient sleep duration seem to confound or mediate that association, and affect lifestyle and the risk of developing weight gain. However, the association of social jetlag with weight gain remained significant after adjustment for multiple variables, including health-related behaviors, socioeconomic factors, obesity and sleep duration, in the current study. This finding suggests that social jetlag may itself influence weight gain status via pathophysiological pathways other than those related to health-related behaviors, socioeconomic factors and sleep duration.

The strength of this study is that it was a large population-based nationally representative study that considered a comprehensive range of possible confounding and mediating factors, including sociodemographic factors, lifestyle and sleep duration.

There were several limitations. The measurements were performed at a certain time in a cross-sectional design; thus, a causal relationship could not be clearly determined. The timing of sleeping and waking on weekdays and weekends were based on a self-report, and are likely to include recall bias. Another limitation was that we did not have information regarding the duration, frequency, or chronic or acute social jetlag over the lifetime. The lack of information on weight change over the lifetime was also a potential limitation. Finally, KNHANES VII did not check body fat composition, and we could not assess the association between social jetlag and adipose tissue mass.

## 5. Conclusions

In conclusion, social jetlag was independently associated with an increased risk of weight gain in men. We propose that social jetlag may contribute to an obesogenic tendency, and should be considered a risk factor. Our findings open a new avenue through which body weight could be managed with a circadian approach. To restore or avoid disruption of the internal rhythmicity, it is time to target behavior modification interventions to reduce social jetlag, including maintaining regular sleeping and waking cycles, and balancing biological and social time. Living ‘against the clock’ may be a factor contributing to the epidemic of weight gain. To stay slim, stay regular. A prospective study is needed to explore the possible causal relationship between social jetlag and subsequent weight gain.

## Figures and Tables

**Table 1:** Characteristics of the study population in men.

	No Change	Weight Loss	Weight Gain	*p-*Value
N (%)	2283 (66.8)	448 (13.1)	686 (20.1)	
Age (years)	48.91 ± 0.412	45.14 ± 0.961	37.0 ± 0.544	<0.001
BMI (kg/m^2^)	24.16 ± 0.076	23.96 ± 0.197	26.19 ± 0.1570	<0.001
≥25 kg/m^2^ (%)	35.5	40.1	60.3	<0.001
Smoking (%)				<0.001
Never	25.1	25.0	32.7	
Past	40.9	31.3	30.0	
Current	34.0	43.8	37.3	
Alcohol drinking (%)				0.002
None	15.2	15.6	9.1	
≤1/week	48.5	51.8	53.6	
≥2/week	36.3	32.6	37.3	
Family income ^a^ (%)				0.330
<200	19.9	22.4	17.7	
200–399	27.2	27.3	30.3	
≥400	52.8	50.4	52.0	
Occupation (%)				0.021
None	23.1	28.6	26.8	
Nonmanufacturing	45.2	42.5	48.1	
Manufacturing ^b^	31.7	28.9	25.1	
Less than high school education (%)	20.0	18.4	9.2	<0.001
Residence in urban area (%)	49.2	51.7	50.1	0.665
Regular exercise ^c^ (yes, %)	48.6	56.9	56.1	0.001
Total energy intake (kcal)	2331.41 ± 23.110	2320.81 ± 56.357	2422.82 ± 50.666	0.259
Carbohydrate intake (g)	337.67 ± 3.330	324.89 ± 7.402	323.27 ± 5.980	0.053
Fat intake (g)	54.33 ± 0.982	56.60 ± 2.398	66.00 ± 2.323	<0.001
Hypertension (%)	34.2	31.7	26.7	0.004
Diabetes (%)	12.4	16.8	6.3	<0.001
Sleep duration ≥7 h (%)	70.5	66.7	67.3	0.209
Social jetlag (%)				<0.001
<1 h	70.1	68.1	52.7	
1–2 h	23.9	23.6	31.5	
>2 h	5.9	8.2	15.7	

Data are expressed as the mean ± SD for continuous variables and as weighted percentages for categorical variables. The comparisons among groups were performed using ANOVA for continuous variables, and Chi-square tests were used for categorical variables. BMI, body mass index. ^a^ Unit is in thousand Korean won/month. ^b^ Manufacturing work was regarded as services, sales, agriculture and fishery industries, and factory work. ^c^ Regular exercise was regarded as ‘yes’ when the subject does aerobic exercise on a regular basis.

**Table 2:** Characteristics of the study population in women.

	No Change	Weight Loss	Weight Gain	*p-*Value
N (%)	2969 (60.9)	568 (11.6)	1341 (27.5)	
Age (years)	50.40 ± 0.447	45.65 ± 0.885	42.70 ± 0.443	<0.001
BMI (kg/m^2^)	22.69 ± 0.081	23.06 ± 0.187	24.68 ± 0.139	<0.001
≥25 kg/m^2^ (%)	21.6	27.0	41.5	<0.001
Smoking (%)				<0.001
Never	91.2	82.9	85.4	
Past	4.7	8.7	8.2	
Current	4.1	8.4	6.4	
Alcohol drinking (%)				<0.001
None	32.0	29.6	25.8	
≤1/week	57.8	56.3	57.4	
≥2/week	10.2	14.1	16.8	
Family income ^a^ (%)				<0.001
<200	24.4	27.3	18.3	
200–399	24.6	28.6	28.4	
≥400	51.0	44.1	53.3	
Occupation (%)				<0.001
None	49.1	48.1	45.5	
Nonmanufacturing	36.9	37.2	45.5	
Manufacturing ^b^	13.9	14.7	9.0	
Less than high school education (%)	31.0	28.8	18.8	<0.001
Residence in urban area (%)	50.5	51.8	51.3	0.879
Regular exercise ^c^ (yes, %)	45.6	46.6	44.5	0.745
Total energy intake (kcal)	1653.65 ± 16.115	1619.38 ± 34.011	1707.32 ± 26.205	0.074
Carbohydrate intake (g)	268.47 ± 2.827	248.41 ± 5.088	262.21 ± 3.853	0.003
Fat intake (g)	38.40 ± 0.629	40.70 ± 1.459	42.38 ± 1.123	0.008
Hypertension (%)	24.9	24.4	17.5	<0.001
Diabetes (%)	10.2	16.7	6.6	<0.001
Sleep duration ≥7 h (%)	67.7	72.2	68.6	0.158
Social jetlag (%)				<0.001
<1 h	68.1	68.0	61.3	
1–2 h	27.1	25.8	30.0	
>2 h	4.8	6.2	8.7	

Data are expressed as the mean ± SD for continuous variables and as weighted percentages for categorical variables. The comparisons among groups were performed using ANOVA for continuous variables, and Chi-square tests were used for categorical variables. BMI, body mass index. ^a^ Unit is in thousand Korean won/month. ^b^ Manufacturing work was regarded as services, sales, agriculture and fishery industries, and factory work. ^c^ Regular exercise was regarded as ‘yes’ when the subject does aerobic exercise on a regular basis.

**Table 3:** Characteristics of the study population according to social jetlag in men.

	<1 h	1–2 h	>2 h	*p-*Value
N (%)	2467 (72.2)	738 (21.6)	212 (6.2)	
Age (years)	49.65 ± 0.445	39.30 ± 0.493	33.24 ± 0.854	<0.001
BMI (kg/m^2^)	24.55 ± 0.080	24.60 ± 0.145	25.21 ± 0.341	0.180
≥25 kg/m^2^ (%)	41.6	42.1	44.9	0.672
Smoking (%)				<0.001
Never	25.5	30.3	26.7	
Past	41.4	31.1	22.1	
Current	33.1	38.6	51.3	
Alcohol drinking (%)				<0.001
None	16.4	9.4	7.0	
≤1/week	47.5	54.4	57.8	
≥2/week	36.1	36.3	35.2	
Family income ^a^ (%)				<0.001
<200	23.1	11.6	18.1	
200–399	25.7	31.9	33.8	
≥400	51.2	56.5	48.1	
Occupation (%)				<0.001
None	28.0	17.3	21.7	
Nonmanufacturing	42.0	54.4	45.9	
Manufacturing ^b^	30.0	28.3	32.4	
Less than high school education (%)	22.2	7.0	9.6	<0.001
Residence in urban area (%)	47.9	53.3	53.0	0.087
Regular exercise ^c^ (yes, %)	48.3	57.5	58.1	<0.001
Total energy intake (kcal)	2304.90 ± 25.441	2425.71 ± 35.194	2489.39 ± 80.351	0.003
Carbohydrate intake (g)	332.43 ± 3.258	337.24 ± 5.130	319.81 ± 9.144	0.241
Fat intake (g)	53.80 ± 1.104	62.22 ± 1.466	70.52 ± 3.874	<0.001
Hypertension (%)	36.8	24.1	19.8	<0.001
Diabetes (%)	13.9	7.5	5.4	<0.001
Sleep duration ≥7 h (%)	66.4	75.5	72.3	<0.001
Weight change (%)				<0.001
None	67.4	58.9	43.8	
Weight loss	13.5	12.0	12.5	
Weight gain	19.1	29.2	43.7	

Data are expressed as the mean ± SD for continuous variables and as weighted percentages for categorical variables. The comparisons among groups were performed using ANOVA for continuous variables, and Chi-square tests were used for categorical variables. BMI, body mass index. ^a^ Unit is in thousand Korean won/month. ^b^ Manufacturing work was regarded as services, sales, agriculture and fishery industries, and factory work. ^c^ Regular exercise was regarded as ‘yes’ when the subject does aerobic exercise on a regular basis.

**Table 4:** Characteristics of the study population according to social jetlag in women.

	<1 h	1–2 h	>2 h	*p*-Value
N (%)	3425 (70.2)	1222 (25.1)	231 (4.7)	
Age (years)	51.54 ± 0.408	41.73 ± 0.470	31.60 ± 0.814	<0.001
BMI (kg/m^2^)	23.55 ± 0.083	22.89 ±0.127	22.71 ± 0.294	<0.001
≥25 kg/m^2^ (%)	30.0	24.4	22.6	0.001
Smoking (%)				<0.001
Never	90.1	87.1	78.3	
Past	5.5	6.6	11.6	
Current	4.4	6.3	10.1	
Alcohol drinking (%)				<0.001
None	34.9	22.0	10.9	
≤1/week	53.3	64.3	72.4	
≥2/week	11.7	13.7	16.6	
Family income ^a^ (%)				<0.001
<200	27.1	14.5	16.7	
200-399	27.0	24.2	26.3	
≥400	45.9	61.3	57.0	
Occupation (%)				<0.001
None	53.4	38.9	30.4	
Nonmanufacturing	33.1	49.8	61.4	
Manufacturing ^b^	13.5	11.3	8.1	
Less than high school education (%)	35.2	13.2	3.7	<0.001
Residence in urban area (%)	48.8	55.1	54.5	0.008
Regular exercise ^c^ (yes, %)	42.5	49.7	57.2	<0.001
Total energy intake (kcal)	1629.17 ± 15.251	1706.07 ± 21.248	1869.39 ± 72.885	<0.001
Carbohydrate intake (g)	265.51 ± 2.490	262.55 ± 3.523	258.47 ±8.900	0.610
Fat intake (g)	37.14 ± 0.598	43.22 ± 0.888	53.63 ± 3.169	<0.001
Hypertension (%)	27.6	14.4	7.3	<0.001
Diabetes (%)	12.1	6.3	2.5	<0.001
Sleep duration ≥7 h (%)	63.8	76.9	80.1	<0.001
Weight change (%)				<0.001
None	60.7	57.6	46.3	
Weight loss	12.3	11.1	12.2	
Weight gain	27.0	31.4	41.5	

Data are expressed as the mean ± SD for continuous variables and as weighted percentages for categorical variables. The comparisons among groups were performed using ANOVA for continuous variables, and Chi-square tests were used for categorical variables. BMI, body mass index. ^a^ Unit is in thousand Korean won/month. ^b^ Manufacturing work was regarded as services, sales, agriculture and fishery industries, and factory work. ^c^ Regular exercise was regarded as ‘yes’ when the subject does aerobic exercise on a regular basis.

**Table 5:** Association between social jetlag and body weight change.

		Fully Adjusted Odds Ratio (95% CI)		
		<1 h	1–2 h	>2 h
Men ^a^	Weight loss	Reference	0.908 (0.677–1.217)	0.975 (0.586–1.625)
	Weight gain	Reference	1.228 (0.963–1.566)	1.787 (1.192–2.679) ^c^
Women ^b^	Weight loss	Reference	0.783 (0.592–1.036)	0.778 (0.460–1.317)
	Weight gain	Reference	0.931 (0.771–1.124)	1.050 (0.694–1.588)

^a^ Data are adjusted for age, sociodemographic factors (family income, occupation and education), lifestyle behaviors (smoking status, alcohol consumption, regular exercise, total energy intake and fat intake), chronic disease (diabetes and hypertension), obesity and sleep duration. ^b^ Data are adjusted for age, sociodemographic factors (residential area, family income, occupation and education), lifestyle behaviors (smoking status, alcohol consumption, regular exercise, total energy intake, carbohydrate intake and fat intake), chronic disease (diabetes and hypertension), obesity and sleep duration. ^c^ *p* < 0.05. CI—confident interval.
